# Minutes and Discussions of the New York Dental Association

**Published:** 1858-03

**Authors:** 


### Proceedings of Societies.

### MINUTES AND DISCUSSIONS OF THE NEW YORK DENTAL ASSOCIATION.

At a meeting of Dentists, held at 57 Bond street, on Wednesday, Nov. 18th, at seven and a half, P. M., Dr. George S. Hawes was appointed Chairman, and Dr. J. G. Ambler, Secretary. The following resolution was adopted:

*Resolved*, That we deem it expedient to have a series of meetings for the interchange of views on subjects relating to the profession.

On motion, it was

*Resolved*, That a committee of one be appointed to make the announcement. Dr. G. H. Perine was chosen.

Accordingly, the following announcement was made: "A meeting of the Dental Surgeons of New York and vicinity will be held at No. 57 Bond street, on Wednesday, Dec. 2nd, at half-past seven, P. M.

"The object of the meeting is to associate, for the promotion of the individual, as well as the general interest of the profession to which we belong.

"The invitation is intended to be general; and it is hoped that no one will feel himself neglected, in case he does not receive a copy of this Circular; but that each and all will interest themselves in the movement, and give it their hearty coöperation and support."

Agreeable to the call, a meeting was held at the above named place, on Wednesday, Dec. 2d, at half-past 7, P. M.

Dr. S. H. Clark was appointed Chairman, Dr. J. G. Ambler, Secretary. The minutes of the previous meeting were read and approved.

After some discussion by Drs. Ballard, McIllroy, Perine, Roberts, Dodge, Covell, and Allen, it was, on motion of Dr. Covell,

*Resolved*, That a committee of five be appointed to present a Constitution and By-Laws.

The committee consisted of Drs. Covell, Ballard, Perine, Allen, and Clark.

On motion, adjourned to meet at same place, Wednesday, Dec. 9th.

Pursuant to adjournment, a meeting was held at 57 Bond street. Dr. S. Covell was appointed Chairman *pro tem.*, Dr. J. G. Ambler, Secretary.

The minutes read and approved. The report of the committee on Constitution and By-Laws was read and adopted.

Dr. Castle thought the Constitution was too democratic This gave rise to some discussion, in which Drs. Ballard, Perine, Clark, and Ambler, took part. Finally, on motion of Dr. Perine, *Resolved*, That we consider the report of the committee, and it was taken up section by section, and re-adopted without debate.

The signatures of members having been appended to the report, the election of officers was proceeded with.

Dr. J. G. Ambler was elected Secretary; Dr. W. B. Roberts, Treasurer—the election of Chairman to take place at each meeting. Business Committee—Drs. C. W. Ballard, John Allen, L. Covell.

Dr. Perine proposed for the evening discussion the "Treatment of Fangs," upon which Drs. Perine, Ballard, Hawes, Clark and Roberts, expressed their views.

Subjects for discussions at the next meeting were proposed, and meeting adjourned.

These meetings are to be held twice a month.

Association met according to adjournment, at 57 Bond st., on Tuesday evening, Dec. 21st, 1857.

Dr. W. McIllroy was called to the chair. The minutes read and approved, after which, the subject propounded by the committee was read, namely, *The clasping natural teeth, inserting artificial ones on plate. The best method of clasping, whether by wide or narrow clasps. The best means of preventing injury to the natural teeth.*

The discussion was opened by Dr. Clark, reading a paper on the subject. He supposed it was safe to say that clasps were always injurious to the teeth. Decay, however, was in a great degree caused not by the action of the clasp, but by the collection of impure matter between the clasp and the tooth, which formed a destructive acid. There was a difference of opinion as to the best width for clasps. His opinion was that in cases where only perpendicular support was wanted, the clasp should be made so as only to touch the teeth in two or three places, and these places as small as practicable.

Dr. Covell considered there was a slight discrepancy in the views presented by Dr. Clark. If, as he stated, teeth were always found to be most sound at the point of contact with the clasp, it would follow that the clasp, instead of being made narrow, should be made to cover every part of the tooth.

Dr. Clark wished to know if Dr. Covell had ever seen a man who could make every part of a clasp touch the tooth, unless he would fill it round and make it regular in shape.

Dr. Covell did not insinuate that they could make the clasp touch every part. He simply alluded to the point that if the places where the clasp touched were the places preserved, then they ought to make it touch as much of the tooth as possible.

Dr. Perine said he had given the subject some thought, as to narrow and wide clasps. If the clasps were narrow, they all knew there was less power of resistance ; where the enamel terminated at the gum, the clasp acted there, the tooth became irritated very *easily*, and inflammation ensued. If narrow clasps were in some respects preferable to wide ones, he would say let them be adjusted so as to come some distance from the gum—as much so as possible—that there might be a stronger enamel to resist the action of the clasp. He explained the method he had adopted in adjusting clasps—leaves quite a space between the plate and clasp, connecting them at two points as high upon the tooth as possible, thus giving a free circulation. Had seen a case recently, similar to the method he spoke of, that was made by Dr. Hale, of St. Louis, which had been worn about twenty years—the gums and teeth were still in healthy condition.

Dr. Ambler asked if Dr. Perine had not found, where clasps were adjusted in the manner he described, the gum to protrude between the clasp and plate.

Dr. Perine replied that he had not, except in cases where the gums were in a soft and spongy state.

Dr. Clark had in his experience found teeth to decay so rapidly under wide clasps, that he was almost ashamed to use them. He endeavored to cover as little of the tooth as possible, and not to touch the neck of the tooth at all. He had seen clasps fitted very handsomely up to the edge of the tooth.

Dr. Perine said he had received a valuable hint from Dr. Crowell in making a perfect fitting clasp, and he would like to hear from him.

Dr. Crowell explained a very ingenious method he adopted, of making clasps fit perfectly to the teeth. Marking the place on the cast where he required the clasp to fit, and causing a deposit of gold or platina by means of a galvanic battery. By this plan he was enabled to deposit a perfect clasp, which would spring over the tooth, and go exactly to the place where he designed it to fit.

Dr. Stratton had been in the habit of using clasps ever since he commenced practice in 1836, and always endeavored to use as narrow a clasp as was consistent with the necessary strength. His wife had worn artificial teeth for twenty-two years, and there was one molar tooth to which a clasp had been fastened all that time, which was good. There were some teeth of so soft a nature that they never should be clasped. In such cases, teeth should be inserted on the atmospheric principle. He endeavored, in such cases, to avoid letting the clasp touch the gum, but brought the clasp as near the end or crown of the tooth as possible, and always had satisfaction in doing so. He never found a tooth to decay quickly when clasped in this manner. Was particular to make his clasp fit as snugly as possible, and explained how he made it from a plaster cast.

Dr. Roberts considered that was the only way that clasps could be made to fit properly. He was opposed to clasping at all. Perhaps he might differ from somo of the profession in this respect; he thought that too many teeth were every year sacrificed by using clasps; that nine cases out of ten, teeth might have been put in without them. It was true there were cases where patients would not pay for an atmospheric plate for one or two teeth, and that they had to do their work, in many cases, according to circumstances, and were obliged to use clasps sometimes where they knew they ought not to be used. The question, as he understood it, was, what kind of clasps had the most injurious effect on the teeth, whether wide or narrow, and of what material. He did not think the material was of much consequence, as long as it was pure gold, silver, or platina, unless there were other materials in the mouth that might affect it. He could not arrive at a certainty which of the two, wide or narrow, injured teeth the most. But one main point was, *cleanliness.* Dentists were not particular enough in instructing their patients, when clasps were used, to remove often and keep the teeth free from food which collects between the teeth and clasps, and destroys the enamel. One other point in clasping was to have the plate so that it would be *perfectly* firm, and in order to do this, they should adopt a clasp according to the nature of the tooth on which it was to hold. There were circumstances that could not be arrived at except by experience and an examination of the mouth.

Dr. Hurd considered the plan a very good one, which was suggested by Dr. Crowell, for obtaining a perfect fitting clasp, but suggested some difficulties in its accomplishments, and asked how he would take the impression of the teeth that were larger at or near the grinding surfaces.

Dr. Crowell replied that he would take the impression with gutta percha—it would yield in removal, and retain the original form of the teeth.

Dr. Dodge explained a method of obtaining a good cast for fitting a clasp, by means of plaster and a little cup of heavy tin foil.

Dr. Ambler said that some remark had been made as to what was necessary to please patients. He thought the subject they had to discuss was one of merit alone—not expediency—not whether it was advisable to do this or that for the sake of the whims of patients. It was simply whether it was best to clasp teeth, under certain circumstances, or not—whether clasps were injurious or not—and if injurious, how did they injure the teeth. With reference to the statement of Dr. Clark, that he never had seen a case where the tooth near the grinding surface was worn by the clasp, he could say also that he had never seen one. His was attributable to the contact of the clasp with the thickest part of the enamel of the tooth, consequently, in his practice, he endeavored to clasp that part of the tooth that would resist friction the most; it was also necessary to preserve a contact with the whole surface of the tooth as much as possible. He believed that teeth were injured more by narrow clasps than by wide ones. Wide clasps wore the teeth but very little. In depositing for clasps, perhaps it was owing to some imperfection in his apparatus, he had entirely failed.

Dr. Dodge remarked he had not made a piece of artificial work in many years; referred to a case where the clasp was connected with the plate at some distance from the edge of the gums, and was worn without injury to the teeth or gums.

Dr. Perine observed that he was glad to know that the method he had adopted was practised by others with so much satisfaction; felt confident, where clasps were used at all, that they should be adjusted on this principle.

Dr. Allen had found the method used and introduced by Dr. Gunning, of swedging plates, entirely successful. He went on at some length to explain the manner in which he managed it. He had almost given up the use of clasps.

Dr. Putnam's experience in the use of all metal clasps had been that the teeth soon became decayed. He had known hard rubber to be used very successfully by Dentists.

Dr. Allen mentioned to the members that they would be pleased or interested in learning that E. A. L. Roberts had succeeded in constructing a very powerful ox-hydrogen blow-pipe, for melting platina—something which the dentists of this city had long wanted. He had melted twenty ounces at one time.

After some further discussion, from which much practical and useful information was elicited, the committee reported for discussion at the next meeting, "The destruction of nerves, when indicated, manner of performing the operation, and subsequent operation on the root;" and meeting adjourned for two weeks.

—

Pursuant to adjournment, a meeting was held at the above named place, January 6th, 1858. Secretary called meeting to order. Dr. Hurd, of Williamsburgh, was called to the Chair. Minutes read and adopted. Treasurer's report was accepted. The subject for the evening's discussion was read and opened by Dr. Bushmore, reading the following paper:

"Much has been said and written upon the treatment of exposed nerves. Many suggestions have been made from time to time, with the hope that they would lead to a radical improvement in the treatment of this class of dental maladies. Still the end has not yet been attained. The object we all have in view, perfection, is not yet within our grasp. And until this is accomplished, attentive and patient investigation not only in this, but in every obscure and perplexing question, should engage every dentist who desires to see his profession rise until it reaches the summit of human skill. The question under discussion, I conceive to be one of the most important which calls for the consideration of the dentist. Simple caries, or those not complicated with disorders of the pulp, no matter where its location, or what its character or extent, may, in most instances, be arrested, and the vitality of the tooth preserved. But caries of a compound character, that which in its progress has reached the pulp, baffles the most consummate skill.

The brain may be deprived of a large share of its nourishment, and yet continue to live and act. Large branches of nerves are excised, and the parts in immediate connexion still perform their usual functions. But once destroy the pulp of a tooth, and sooner or later, it is attacked by disease, which, mocking at our remedies, proves its destruction. Now, why is this? Is the office or function of the pulp of a vital character, and thus absolutely necessary to the life of a tooth, so that its destruction, no matter by what process, is a death-blow; or is the failure in these cases to be looked for in the treatment pursued?

Either the presence and healthy condition of the pulp is essential to the vitality of a tooth, or it is not. If the former, then to destroy the pulp is to destroy the tooth. If the latter, then is not the present method of treatment erroneous?

I incline to the opinion that the latter is the case. That the pulp is not of vital importance, and as a sequence to the opinion, that the employment of arsenic is highly injurious. Is not its application almost invariably followed by periostitis of a more or less violent character. Arsenic possesses the power of depriving animal tissue of its principal property which appertains to its vital condition, viz: that of suffering and effecting transformations, or, in other words, organic life is destroyed. Consequently this periosteal inflammation does not result from the effect of free arsenic, but is produced, I think, by reflex action. The reflex action of nerves is capable of transmitting all kinds of expressions, or shocks to remote organs. It can not be difficult, therefore, to conceive how the effects of an injury, such as is caused by arsenic on the pulp, may influence the vital properties of the periosteum, so as to derange its functions, and thereby constitute a local irritation in that part, and it is a maxim, that where there is irritation, there will be increased vascularity, which in time sets up an inflammatory action. This inflammatory action, it is true, may be combatted, and, for the time being, may be subdued by local and constitutional treatment. but my faith in complete and ultimate success is very weak.

I have pursued the usual method of treatment, the application of arsenic, removal of the pulp, and the subsequent filling of the roots; but I can not call to mind a single instance in which the tooth has not given unmistakeable evidences of periosteal inflammation. I do not judge wholly from my own experience, for that is in a measure limited. I may also have been at fault in performing the operations, but I have my opinion upon the evidence and experience of others, in connexion with my own. I throw out these thoughts with the hope that they may call forth remarks for my enlightenment."

Dr. McIlroy remarked on the importance of the subject, and was listened to with much interest—spoke of teeth in his own mouth that had been treated and the roots filled; also presented interesting specimens of teeth that the nerves had become ossified.

Dr. Perine observed that it had been remarked by some member at the Convention at Boston, that Dentists were disposed to ride a "Hobby." Said he was glad that this mode of operating was with him a Hobby, and that he should continue to ride it as long as he was successful in the practice. His method of filling the roots of teeth corresponded with the mode introduced by Dr. J. S. Clark, of New Orleans. He believed it all-important to save the vitality of the pulp, but when it became necessary to extirpate the nerves, he preferred to remove, by mechanical means, when it was possible to do so, but he found his patients would not always submit to the operation. He treated abscess with creosote—also nitrate silver.

Dr. Hawes asked how he removed the nerve when the cavity was smaller than the brouch used, as was sometimes the case.

Dr. Perine replied, in cases where it was impossible to extirpate the nerves with the brouch, he enlarged the nerve cavities, but preferred the natural shape of the canal. There were cases, however, in his practice, that he found it almost impossible to remove it entirely.

Dr. Covell remarked, that he had not practiced to much extent the extirpation of the pulp and filling the roots of teeth—preferred saving the vitality of the nerve—had been successful in capping the nerve and filling sensitive teeth by a cover of finely pulverized charcoal. He referred to cases in which he had filled teeth in this manner, a number of years since—had seen them quite recently—found them in a healthy condition, and had been free from pain.

Dr. Perine asked if he had noticed any discoloration in the teeth from the coal.

Dr. Covell replied he had not—the coal was very finely pulverized and placed in the bottom of the cavity. Got the hint from one of the profession in Philadelphia, a number of years ago.

Dr. Roberts remarked, in using a substance as a non conductor, would recommend horn or bone.

Dr. Keys thought it highly important, in preparing the nerve cavity for filling, that the entire portion of pulp be removed, to preserve the health of the tooth. He believes that no ossification can take place without inflammation—thinks it does not depend so much upon the perfect filing of the root as to prevent the inflammatory action.

Dr. McIllroy asked why it was that aged persons' teeth ossified without ulceration.

Dr. Keys replied, that the inflammation did not go so far as to produce ulceration.

Dr. McIllroy said he would like to hear from Dr. Lovejoy, who had a large experience in the treatment and filling of roots.

Dr. Lovejoy does not profess to have had so large an experience as some others. He did hope to hear more upon the treatment of abscess. His practice is to probe the internal membrane and the sack, if possible, remove the matter—uses creosote, nitrate silver—leeches gums freely. After bringing about a healthy action, fills the roots—avoids pressing air or moisture in advance of the filling.

Dr. Putnam said he had retired from the labors of the operative department, and was glad he had done so, but he would say a few words. Has never practised root filling.

Spoke of filling the apex and leaving the canal open, and completes the operation by plugging the crown cavity. Treats abscess with creosote. Thinks much of the practice of drilling the root as recommended by the late Dr. H. and others. If the cavity of the tooth thus treated be kept open, there is no trouble. Has a tooth treated thus in his own mouth. Has been successful in this mode of treatment.

Dr. Roberts said, there were many theories advanced in the practice of Dentistry, and it was of vital importance to patients that correct conclusions should be arrived at. He, therefore, thought Dr. Putnam's mode a bad one, and had a tendency to mislead young dentists. He had practised this to some extent, but had abandoned it; he could see no necessity for drilling the tooth and leaving an opening to be constantly discharging, when a filling could be removed and the root properly treated and cured permanently. He said there were three important facts to be kept in mind in the filling of badly decayed teeth: 1st, always preserve the nerve alive if possible; 2d, when impossible, it should be removed and the tooth filled to the apex of the root. He had no hesitation *in saying*, that nine cases in ten could be saved; 3d, when the disease had progressed to ulceration, then proper remedies should be applied, and tooth filled as before spoken of. He decidedly objected to the wholesale extracting of teeth when the nerves are exposed or abscesses formed.

Dr. Clark said he was glad to hear the old practice once more spoken of. There was a time he practiced it, but it was to give immediate relief. Thinks if a nerve can be covered it would be better. Would caution younger members of the profession to be careful about the destruction of nerves. His method of filling nerve cavities is to roll the gold foil in the form of a pin and carry it to the apex with a small instrument for the purpose. Was careful not to force air in advance of the plug. He loses more than one in ten after the filling of the roots.

Dr. Perine remarked, he had never resorted very extensively to the drilling the roots of teeth. Thinks in such cases, after treatment, the root should be filled.

Dr. McIlroy said he had been much gratified with the discussion of the evening; he hoped hereafter to hear more on the canthology of the teeth.

The time having expired allotted for the discussion, the committee presented the subject for discussion at the next meeting, when, on motion, adjourned to meet two weeks from this evening.

---

The New York Dental Association held its regular meeting in Bond street, on Wednesday evening, Jan. 20th, at 7½ o'clock. Dr. Lovejoy in the chair.

The subject for discussion was announced to be, *The treatment of diseased teeth and gums, which have become so from other causes than actual decay of the teeth.*

Dr. Ambler said they were all aware of many cases of diseased gums, which were apparently causeless. Most of them were due, in their incipient stages, to the accumulation of tartar; but he had come in contact with many cases where there was not the least appearance of tartar, and the gum at the edge was apparently healthy; yet the teeth were easily loosened, and in some instances unhealthy matter discharged at intervals. He cited one or two examples of cases that had no apparent origin or external cause. It was about four or five years since the first really marked case came under his notice, and which he had almost entirely cured, simply by separating the gum from the tooth to a certain point, probing it as far as possible, and inserting the instrument between the gum and the alveoli, and adopting the principle of a seton, made by a small linen or silk cord, saturated with nitrate of silver. He had introduced this in several instances, where it had seemed to concentrate the disease. Accompanying this, he used a wash of solution of tannin. There were other cases, which owed their origin to tartar, where he had removed the tartar, and had been able to retain the tooth for years with a simple solution of tannin, myrrh, and muriate of zinc; afterwards using a wash with a stronger solution of tannin. In reply to a question whether, in the cases he first mentioned, the patients had ever been salivated, he stated that in one case he had, and in the other, not. The gums indicated a dormant state of inflammation. There was a lack of vitality about them, although no ulceration, or anything to indicate a seated disease. It seemed to be, more than anything else, a looseness of the teeth at times. On extracting a tooth in one case, he found that the nerve was alive and healthy—there appeared to be no disease of the nerve, but it seemed to be a dormant, inactive state of the gum, which produced a loosening. He remarked, too, that the gums were not red nor sensitive;—there was an almost entire absence of sensibility.

Dr. STRATTON had, in his practice, seen many cases where teeth became loose from causes unknown to him, and had treated them somewhat in a similar way to that described. He always made use of a weak tincture of myrrh, with plenty of tannin in it. He recollected one instance in which he had to cut into the alveolar process one to two inches in length.

Dr. HURD had heard of cases somewhat similar to that described by Dr. Ambler; and he made up his mind there was no such thing as curing them. One case he had was that of a young man whose teeth he found somewhat coated with tartar. He advised him to brush his gums thoroughly, and gave him a wash of myrrh; but it did not seem to have the desired effect. He commenced lancing the gums, and used to bleed them sometimes twice a week. The gums were not sensitive. He had thus succeeded in reducing the gums, so that they became perfectly healthy, apparently. The patient went away for a year, and on seeing him again, he found the same difficulty had returned. After long treatment, he concluded it was something constitutional, and that he could not remedy it.

Dr. McILLROY was afraid they had got hold of a rather impracticable subject. The natural divisions of it would seem to be into loose teeth, as produced by constitutional, or local causes. When produced by a local agency, they all knew pretty much what remedies to apply—principally to scarify the gums and keep them clean. When produced by a constitutional cause, much had been said and written as to it, but there was nothing satisfactory. Some writers traced the loosening of teeth to a great many causes; some to long-continued mental emotion, and some to consumption; it had been said, too, that in cases of consumption, loosening the teeth became a counter-irritant, and arrested the disease. He never saw much effected by any mode of treatment, where the alveolar process was lost—indeed, he never saw the first case of recuperation of the alveolar process. A wash which he had found to do some good to diseased gums was made by burning copperas for three hours, to a powder, and adding water to it. Altogether, the subject of loosening of teeth, from constitutional causes, was one, he thought, which belonged rather to physicians than dentists.

Dr. Ambler asked if Dr. McIllroy had ever noticed a case where the gums were apparently healthy, and yet the teeth were loose in the socket, independent of the administration of mercury? He replied that he had heard of such.

Dr. Putnam objected strongly to the use of charcoal as a dentifrice. He was able to detect its use even at a period so far back as ten years by its effects on the gum. He had frequently seen cases where the gum, though apparently healthy, had receded from the neck of the tooth, which he attributed to the use of charcoal, which, being like diamond dust in its nature, cut and irritated the gum.

Dr. Stratton's experience differed from Dr. Putnam's. He had never seen the teeth denuded (if he might so express it) by the use of charcoal as a dentifrice. He also believed that chewing tobacco was not at all injurious to the gums and teeth, as some supposed. Smoking had a tendency to harden the teeth. He never knew a great smoker but what had a hard dentine. A number of his patients used Scotch snuff to clean their teeth, and he found their gums were very healthy and in good order. He knew one lady whose teeth had been very loose, and the use of Scotch snuff as a dentifrice made her gums healthy and sound.

A MEMBER suggested that if ladies knew how offensive such a dentifrice made their breath, they would scarcely be willing to use it.

Dr. DODGE inquired if members had found that unhealthy gums prevailed more in males or females, between the ages of fifteen and forty-five. It seemed to be the general experience that disease of the gums prevailed most with females, during the periods of gestation and lactation.

In reply to a question from Dr. Dodge, as to what he would do with a case where the gums were spongy and the teeth beginning to get loose—whether first remove the tartar, or first apply an astringent,—Dr. ROOT replied that he would first scrape the entire tooth, where the tartar was, and then apply washes,—because his opinion was that the tartar was the first cause of the trouble. The gums would always recede from tartar.

THE CHAIRMAN believed that there were in some constitutions a predisposition to disease in the membranes of the teeth. There was no reason why scrofula, for instance, should not manifest itself in the membranes around the teeth, as well as in any other part of the system. He related the case of a lady who called upon him about thirty years ago. Her teeth appeared to be very clean, but they were held in by little more than the gum in the socket. The alveolar process had the appearance of being absorbed to a considerable extent. Her teeth were perfectly sound in every direction, and yet became so loose, that in the course of fifteen years they had *all* to be pulled out. Then the gums healed up, and appeared healthy. Now, he was confident this was not produced by any external or local cause that was perceivable; but was a constitutional difficulty. Where there was tartar on the teeth, and the gums diseased, he was in the habit of using a solution of muriatic acid, to loosen the tartar, before scraping it off, and it also produced an alterative action, which was beneficial. Where the tartar was very old and strong on the tooth, he used the muriatic acid pretty strong.

Dr. BURRAS had for many years practised in a very dirty section of the city, where he had ample opportunity of observing the worst descriptions of diseased gums—in most cases produced by neglect and dirt. He recommended to people of that description the use of anything that would create *friction* and some sort of *action* in the mouth—the chewing of tobacco or anything else. Constant application of the brush and cold water was the great preventative and cure for unhealthy gums. To establish his opinion that diseased gums arose in many cases from causes distinct and remote from the mouth, he stated the case of a lady whom he treated, whose mouth had become very sore from an interruption in her menstrual discharges. He gave some remedy to cure that difficulty, and her mouth got entirely well.

Speaking of the soundness of some roots, Dr. STRATTON related a remarkable case within his own experience, of a man who wore four pivot teeth for thirty-three years.

Dr. HURD followed by an anecdote of a lady who had in a pivot tooth for fifteen years, which had never in that time loosened, so that she became herself really forgetful of the fact that it was not a natural tooth.

Dr. DODGE'S opinion, after a few years of practice, was that there were few cases of diseased gums which were not caused by dead teeth, or by uncleanness. Dr. Burras had spoken of a dirty section of the city. He was sorry to say that, in respect to cleaning teeth, all New York was closely related to that section.

There were, undoubtedly, some cases where disease was caused by early bad treatment, or by something wrong in the constitution; but in the great majority of cases, where the brush was *carefully* used once or twice a day, the gums never became diseased round living teeth. One instance might prove a useful illustration to young dentists. A young lady whose gums were diseased, called on him some years since.

Her teeth were tolerably clean; he found but little tartar; she used her tooth-brush and powder every day, she said, from five to ten minutes. He desired her simply to go home, and for one month, when cleaning her teeth every day, lay her watch beside her for *five* minutes, and brush her teeth carefully and faithfully for that time, and to let him see her again. She promised to do so. At the end of a month she returned, with her mouth perfectly healed, and thanking him, said, "Doctor, I never knew how long five minutes was before. I have from childhood only given an excuse for a cleaning to my teeth." It was not only necessary to brush the teeth (and this fact should be impressed on patients), but that we should give them *enough of it;* take the right kind of brush, and a wash if necessary, many of which were good in their way, but the chief thing needed was constant and careful friction with the brush.

Adjourned to Wednesday evening, Feb. 3d.

—

THE NEW YORK DENTAL ASSOCIATION met pursuant to adjournment, on Wednesday evening, Feb. 3d. Dr. Clark in the chair.

After the usual preliminary business, the secretary announced the subject for discussion: *What is the best material for the basis of Artificial Dentures?* In the discussion of which question, the whole subject of the materials of teeth and the manner of inserting them was to be embraced.

Dr. MAIN said it would take a long time for the practitioner to explain every way he had gone through in making artificial dentures. For himself, he had tried almost everything, and he went into an enumeration of the various materials which had been experimented upon from the earliest day up to this time. He had tried the continuous gum, in blocks and in full sets, on platina; but the objections to this mode he considered numerous, the chief being that the work was too heavy, had no elasticity, and the mouth sweated under the effect of the platina plate, so that there was constantly a leaking of saliva to be sucked from the rear part of the plate. From the introduction of vulcanized rubber, he believed much benefit would be yet derived; but he had, after trying all the systems, come to the conclusion to go back to the first principle of a gold plate, with single gum teeth well ground and fitted upon it. This he had found, after long experience, to give most satisfaction to the patients.

Dr. HURD had tried the tin basis with very great success. With reference to platina work, he had not used it as much as a great many others; still he had seen nothing in artificial dentistry, so neat, natural, and easy to the mouth of patients, as sets made on platina. With respect to the sweating of the mouth, he considered it would do so under any plate. One thing greatly in favor of platina was, that there was no chance of deposit from it. Dr. H. gave an instance of a lady who had worn a set of teeth on a gold plate for twelve years, and for whom he afterwards made a set, with continuous gum, on platina. After two or three years' experience with the latter, she pronounced them the perfection of dentistry, and added, that one thing she knew, there was no metalic taste, and no smell about them when taken out of the mouth.

Dr. ALLEN considered that in the construction of artificial dentures, it should be their aim to approximate as much as possible to the natural organs of the mouth. There were six important considerations that should govern them in this branch of the profession:

First. The materials used should be incorruptible, and the denture so constructed as not to admit of vitiated secretions.

Second. A perfect adaptation of the denture to the mouth.

Third. The restoration of the natural form and expression of the mouth and face, in cases where the muscles had become sunken.

Fourth. A natural form should be given to the lingual surface of the denture, in order that the tongue might articulate with ease and clearness.

Fifth. Sufficient strength to subserve the purposes for which they are intended.

Sixth. Artificial dentures should be so truthful to nature in appearance as to elude detection by the common observer.

All of these points, besides others of minor consideration, he claimed for continuous gum work. As regarded the first point, the plates were pure platina, the solder pure gold, and the teeth, body, and gum enamel all composed of pure minerals and metalic oxides. Platina, besides being pure, had sufficient ductility to be swaged perfectly to a cast from the mouth, and had much less expansion and contraction, when subjected to extreme transitions of heat and cold, than any other metal used for dental purposes, being only 1 in 1176, while that of 18 carat gold, is 1 in 600. Its capability of resisting the most intense heat of the furnace, was another reason for its adoption, as they could fuze the compound for continuous gum upon it without the least danger of melting the plate. Again, it had a much greater affinity for silicates than gold. Dr. A. proceeded to give his views as to the best manner of "articulating" a set of teeth. He next called attention to the manner of restoring the natural form and expression of the face, where it had become sunken. There were four points of the face which, in many persons, the mere insertion of the teeth would not restore, viz: one upon each side, beneath the malar bone, and one upon each side of the nose, on a line towards the molar bone. the muscles which form these portions of the face, he raised by means of attachments formed upon the denture, of such shape and size as the case might require. In doing this, the operator should study the face of his patient as would an artist in taking a picture, making him laugh and talk, so as to get the natural expression. Finally, he said, he preferred the continuous gum, because he could select just such teeth as might be requisite for any particular case, having especial reference to the shape, form, length and character of the teeth, which should be in perfect harmony with the other features of the patient, and because, when mounted upon the plate, they were able to fill up all the interstices in and around the base of the teeth with a silicious compound, perfectly pure and unobjectionable, in the mouth, and which formed an artificial gum, truthful in appearance, and also the natural roof of the mouth; because they could rejuvenate the face. He had thoroughly tested it for the last seven years, and was convinced that its accession would give great prominence to the artificial branch of the profession.

Dr. PUTNAM had known plates of sole leather to be made for full sets of teeth, and, according to accounts given by the "shoemaker dentist," they answered an excellent purpose. He informed him that he was particular to obtain the oak-tanned leather, as its astringent qualities were so great that the best gum tinctures bore no comparison in keeping a healthy action in the mouth, and besides, said he, "a plate of leather is *bound* to stick." His most approved plan was to obtain a plaster model, wet the leather, and keep it pressed upon the cast until dry; the teeth were arranged and rivited to a continuous lining formed from a narrow strip of leather, which he afterwards sewed through and through to the plate. His *principle* Dr. P. considered a good one, in endeavoring to dispense with metals. So far as an experience of eighteen years enabled him to decide, he should say that they ought to be decided against the use of any material in the mouth (if a better one could be obtained,) which, in one case in ten, would oxydize, produce galvanic action, or become offensive from secretions. His experience in the case of gold plates did not differ, perhaps, from that of the majority of dentists. He had found, however, that single, plain teeth, well fitted, made a more cleanly piece of work, or single gum teeth, and quite as serviceable. Of the two latter he had never been able to determine which would be most durable and least liable to accident. Of the theory of 18 or 20 carat gold producing galvanic action in the mouth, he knew that his views would not be endorsed by all; but, being in possession of well-attested cases, certified to by physicians and chemists, and proved by the patient laying aside the plate, he was confirmed in the belief that many cases did exist which were often attributed to other causes. So far as the purity of metal goes, and so far as any electrical effects in the mouth are concerned, there was no doubt that platina stood preëminent; but the yielding nature of platina proved fatal to partial pieces. Dr. P. considered that he had overcome most of the objections which attached to metallic plates and the continuous gum, by the use of *Vulcanite Rubber*, which he invariably used as a base for pieces from one tooth to full sets, without the least sign of the material giving way, or changing by the action of fluids or other causes in the mouth. In its use, he had not one case in a hundred to repair from the accident of a broken tooth. He invited members to test it in their practice, and to aid in prosecuting experiments to improve its color, which was very dark, and otherwise overcome obstacles presented in its manipulation.

An old gentleman was present who had worn a full set of teeth on Vulcanite Rubber base for two years, which appeared in very good condition. One tooth, which had been broken by a fall, was replaced, Dr. P. said, simply, by sawing out the piece, putting in another tooth, with a fresh piece of rubber, and subjecting the part to a vulcanizing process.

Dr. Laroche stated that he had had very good success in platina work, with continuous gum. He had made over two hundred cases, out of which he had seen but one piece crack, and not one plate give way. He was very careful in backing to let the linings overlap, so as to make a solid piece all the way round against the teeth, and to this, with great care otherwise, he attributed his success and the lasting character of the work.

Dr. Porter, in answer to a question, whether porcelain teeth were as strong after having been put upon a platina plate, and baked in continuous gum body, as before, answered, that he thought they were not, as in the process of baking they absorbed a portion of the flux.

Dr. MAIN believed the teeth were materially injured by the process of baking in the continuous gum. He had made many sets with continuous gum, and found that the trouble and cost of repairing was greater than the original making of the work.

Dr. FRANKLIN had no doubt that Dr. Main was sincere in his opinion; but he could not but feel that his ill-success was owing, not to any fault in the composition of continuous gum, but to want of proper care and workmanship, since he (Dr. F.) and many others, who had given the matter careful consideration, had been most successful in the results produced. He had seen how, either from want of knowledge, or proper care, nineteen-twentieths of the continuous gum work made by some men was botched; but, again, where dentists had made themselves fully acquainted with the materials and manner of using them, he affirmed, that work had been produced, most satisfactory in its character, capable of bearing the greatest amount of lateral pressure, and most nearly resembling the natural gum. He had been a long time engaged in the manufacture of continuous gum, and in over two years he had not known a single piece to fail from any cause. Great care, however, was to be observed, in giving the proper amount of heat in baking.

Dr. ROBERTS said, that in reference to the observation made by Dr. Porter, that he considered the porcelain teeth weakened in the process of baking, by absorbing a portion of borax; he would mention that in the body now used borax was dispensed with.

Dr. MAIN asked what was substituted for borax?

Dr. ROBERTS said that was something which his brother, who made the new body, kept a secret.

Dr. ALLEN said that a very important point to be attended to in making the continuous gum, and preserving the strength of the teeth, after baking, was a proper and careful process of annealing. Something had been said as to the unyielding nature of this description of work, but he did not consider there was any necessity for more yielding than the natural gum afforded.

Dr. CLAY had used the continuous gum work for six years, and found less failures in it than with any other style of work.

Dr. PORTER, in answer to a question as to which description of work he was called upon to do most repairs, answered, that, in proportion, he had to do less repairs upon single teeth, on a gold plate, than upon block, or other descriptions of work.

Dr. ROBERTS said that Dr. Hawes and himself had frequent discussions in regard to the strength of teeth after being put up in continuous gum work. When they first commenced that description of work, the teeth made were very frail, so that, in many instances, after being baked on the platina plates, they could crumble them off with the fingers. That was a great objection, which, however, he thought, had been since overcome by the great improvement in the making of teeth. He considered that artificial dentistry was a branch of the profession which had been very much neglected; or, at least, the profession did not seem to feel the responsibility that rested upon them in regard to inserting artificial teeth, after the natural ones had been lost. In doing this, there were several important points to be considered: First. What has been lost? Second. What were they required to restore? How were they to restore it? and with what material? These were questions which suggested themselves to any practical dentist, when his services were required to perform an operation of artificial dentistry. Let us examine and see what has been lost. First. The teeth, next, the alveolar process; then resulted difficulty of speech, and always a change in the natural expression of the face. These it was the dentist's duty to restore with the best material and in the best manner possible. By the old style of work, they could restore the teeth, and partially the alveolar ridge and speech; but they could not, in every case, restore the expression of the mouth and face. In this, which he might call *artistic* dentistry, there was something more required than mere mechanical skill. There were also two other very important points to be observed—cleanliness and durability. He contended that he knew of no process so complete as work performed with continuous gum, by which he was enabled to treat successfully all the important points. Yet it was certain there was no work introduced into the profession that had been, in many instances, so carelessly thrown together. The great difficulty had been from ignorant manipulations at the furnace, and the lack of information among members of the profession in regard to silicious compounds. Where the work was put up with all the latest improvements, they could obtain every thing that was required of artificial teeth.

Pending Dr. Roberts' remarks, the hour for adjournment arrived, and the same subject was noticed to be continued at the next meeting.

